# Oxidative modifications of foetal LDL-c and HDL-c lipoproteins in preeclampsia

**DOI:** 10.1186/s12944-018-0766-9

**Published:** 2018-05-10

**Authors:** G. León-Reyes, S. Espino y Sosa, R. Medina-Navarro, A. M. Guzmán-Grenfell, A. X. Medina-Urrutia, S. Fuentes-García, G. J. J. Hicks, Y. D. Torres-Ramos

**Affiliations:** 1 Departamento de Inmunobioquímica, Instituto Nacional de Perinatología, Secretaría de Salud, Montes Urales 800, Miguel Hidalgo, Lomas Virreyes, 11000 Ciudad de México, Mexico; 2 Subdirección de Investigación Clínica, Instituto Nacional de Perinatología, Secretaría de Salud, Ciudad de México, Mexico; 3 Departamento de Metabolismo Experimental, Centro de Investigación Biomédica de Michoacán (CIBIMI-IMSS), Morelia, Michoacán Mexico; 4 Instituto Nacional de Cardiología, Secretaría de Salud, Ciudad de México, Mexico; 5 Comisión Coordinadora de los Institutos Nacionales de Salud y Hospitales de Alta Especialidad, Ciudad de México, Mexico

**Keywords:** Lipoproteins, Newborn, Oxidative damage, Preeclampsia, Lipids, Lipoperoxidation, Paraoxonase-I, Antioxidants

## Abstract

**Background:**

Oxidative modifications have been observed in lipids and proteins in lipoproteins isolated from women with preeclampsia. Thus, newborns could also be susceptible to this damage directly through their mothers. In this study, we evaluated the oxidative profile of LDL-c and HDL-c lipoproteins isolated from the umbilical cord from newborns born to women with preeclampsia.

**Methods:**

Thirty newborns born to women with preeclampsia and thirty newborns born to women with healthy pregnancies were included. Lipid-damage biomarkers, including conjugated dienes, lipohydroperoxides and malondialdehyde, were measured. The reduction of nitroblue tetrazolium, formation of dityrosines, and carbonylation of proteins were assessed as indicators of protein damage. The protective activity of paraoxonase-I on HDL-c particles was evaluated. The total antioxidant capacity and lipid profiles were quantified in plasma. Data were analysed using Student’s t-tests and Pearson correlation coefficients.

**Results:**

Compared with the control group, the preeclampsia group had an increase in the percentage of lipid damage in both lipoproteins. There was an increase of 23.3 and 19.9% for conjugated dienes, 82.4 and 21.1% for lipohydroperoxides, and 103.8 and 51.5% for malondialdehyde in LDL-c and HDL-c, respectively. However, these infants did not show evident damage in protein oxidation. The activity of the enzyme paraoxonase-I was decreased by 36.2%; by contrast, the total antioxidant capacity was increased by 40% (protein) and 28.8% (non-protein).

**Conclusions:**

The oxidative modifications that occur in HDL-c and LDL-c isolated from newborns from women with preeclampsia are mainly caused by lipoperoxidation processes related to evident paraoxonase-I inactivation. The absence of protein damage is likely linked to an increase in total antioxidant capacity. Therefore, antioxidant support could be helpful in reducing oxidative stress in mother/newborn dyads.

## Background

Preeclampsia (PE) is a multisystemic syndrome defined by arterial hypertension and proteinuria developing after 20 weeks of gestation [1]. This syndrome affects approximately one-third of all pregnancies [2] and causes significant maternal, fetal and neonatal morbidity and mortality [3].

During healthy pregnancy, there is an increase in oxygen demand coupled with an increase in the production of reactive oxygen species (ROS) and reactive nitrogen species (RNS) to carry out signaling for physiological processes in pregnancy, such as oocyte maturation, ovarian steroidogenesis, ovulation, implantation, blastocyst formation, luteolysis and luteal maintenance [4, 5]. Preeclamptic women can present associated endothelial dysfunction, dyslipidemia and exacerbated systemic production of free radicals [6]. Specifically, our working group previously demonstrated that low-density lipoprotein-cholesterol (LDL-c) and high-density lipoprotein-cholesterol (HDL-c) in preeclamptic women increase both lipid and protein oxidation products, which has been associated with the intensity of damage. Conjugated dienes (CDs) (mild), lipohydroperoxides (LHP) (moderate) and malondialdehyde (MDA) (severe) are markers of lipoperoxidation. Reduction of the NBT compound (NBT reduction) (mild), production of tyrosine dimers (DTs) (moderate) and carbonylation of proteins (CP) (severe) are markers of protein oxidation. These findings are of great biological importance since these factors can cause biochemical changes to lipoproteins that lead to vascular endothelial dysfunction and create maternal complications that are characteristic of PE [7].

Circulating LDL-c guarantees a constant supply of cholesterol for tissues and cells; cholesterol is required for membrane synthesis, modulation of membrane fluidity and regulation of cell signaling pathways. LDL-c particles are highly heterogeneous in nature and vary in density, size, surface charge and chemical composition; these biochemical characteristics determine the fate of LDL-c in the subendothelial space. Small and dense LDL-c particles are more atherogenic than their light counterparts. The main modifications of LDL-c occur through oxidation, enzymatic degradation or lipolysis. Modified LDL-c accumulates in the intima of the arterial wall where apo-B100 binds to proteoglycans of the extracellular matrix through ionic interaction. As a consequence, LDL-c becomes trapped in the subendothelium, where it is prone to oxidation processes and aggregation, promoting rapid uptake of these particles by macrophages to form foam cells. These alterations depend on LDL-c metabolism, which is predominantly triggered by molecular changes in LDL-c; it is of paramount biomedical importance to explore the structural features of LDL-c particles in great detail [8]. Moreover, HDL-c transports excess cholesterol from the blood to the liver for excretion or utilisation (reverse cholesterol transport). Even HDL-c has anti-thrombotic, anti-coagulant, anti-inflammatory and anti-atherosclerotic properties by accepting cholesterol from lipid-laden macrophages and stimulators of the production of nitric oxide. HDL-c possesses the PON-I enzyme, which provides important, protective antioxidant activity by hydrolysing LHP formed during lipid oxidation, thus protecting it from oxidative damage induced by LDL-c, HDL-c and other biomolecules. However, during chronic oxidative stress (OS), the function of HDL-c can be affected, leading to inactivation of the PON-I enzyme. The consequences include loss of normal biological functions, induction of the proinflammatory state, adhesion of macrophages to the vascular wall, an increase in atherosclerotic lesions, and other complications [9].

In addition to maternal damage caused by OS during PE, newborns could be susceptible to this damage directly through their mothers because elevated ROS/RNS and maternal oxidised lipids, such as fatty acids and cholesterol, can cross the placenta, representing a direct influence on fetal lipids [4]. In addition, neonatal antioxidant mechanisms are not fully developed, making neonates more vulnerable [10]. Therefore, it could be inferred that the oxidative process present in maternal lipoproteins can occur in fetal lipoproteins. These phenomena have not been detailed in newborns and could contribute to neonatal complications at birth such as low birth weight, prematurity, retinopathy, anemia, and other neonatal disorders that have been categorised as fetopathies induced by PE [11]. Therefore, in this study, we evaluated the oxidative profile of lipids and proteins that comprise fetal LDL-c and HDL-c lipoproteins from the umbilical cord of newborns from women with PE.

## Methods

The aim of this study was to evaluate the mechanisms of lipid and protein oxidation in LDL-c and HDL-c lipoproteins isolated from the umbilical cord of newborns from women with PE. The design of this study was transversal and observational.

### Patients

Sixty newborns were included in this study and distributed into the following groups: 30 newborns from women without PE (control group) and 30 newborns from women with PE. All newborns had a gestational period of 35–40 weeks. The inclusion criteria for the control group were newborns from normotensive women that experienced a normal course of pregnancy according to clinical and ultrasound findings. The inclusion criteria for the PE group were newborns from women who matched the diagnostic criteria of the American College of Obstetricians and Gynaecologists for PE with severe symptoms [1], including blood pressure ≥ 140/90 mmHg and proteinuria ≥300 mg, or in absence of proteinuria, any of the following conditions: thrombocytopenia, renal insufficiency, impaired liver function, pulmonary edema or cerebral or visual symptoms. The following exclusion criteria were used for both groups: newborns who presented with infection or sepsis; malformations diagnosed at birth; metabolic diseases; and/or mothers with gestational diabetes mellitus, cardiovascular, autoimmune, renal or hepatic disease and/or received pharmacological treatment that interfered with lipid levels. All the newborns were born in the National Institute of Perinatology (INPer) in Mexico City. Rigorous selection, recruitment and collection of biological samples were performed by clinicians of the INPer. All women were informed of the goals of this study and provided their written consent prior to participation in the study. The study was performed according to the principles outlined in the Declaration of Helsinki. The INPer committee for research, ethics and biosafety approved the protocol (212250–3210–21,001-02-14).

### Sample size

We calculated the appropriate sample size using the mean difference formula, and we used the plasma levels of LHP in normotensive and PE women as references [12]. The calculated sample size was 17 newborns for each group, and we included 30 newborns in the control group and 30 newborns in the PE group.

### Biological samples

We used 5 mL of blood samples from the umbilical cord artery collected in tubes with EDTA (BD Vacutainer, USA) after delivery. Biological samples were centrifuged at 2500 rpm for 15 min to obtain plasma. Plasma was stored at − 70 °C until analysis of the lipid profile, biochemical tests and isolation of lipoproteins.

### Laboratory procedures

In the plasma lipid profile, total cholesterol (T-Chol), triglycerides (TAG), HDL-c, Apoprotein-A (Apo-A) and Apoprotein-B (Apo-B), including glutamic oxaloacetic transaminase (GOT) and glutamic pyruvic transaminase (GPT), were measured on a Hitachi 902 autoanalyzer (Barcelona, Spain. Boehringer Mannheim, S.A) using commercially available kits (). LDL-c was estimated using the Friedewald formula as modified by DeLong [13].

### Isolation of lipoproteins

Lipoproteins were isolated from plasma at a density of 1.21 g/mL for HDL-c and 1.063 g/mL for LDL-c via sequential preparative ultracentrifugation in a Beckman TL-100 ultracentrifuge at 4 °C. HDL-c was dialyzed against phosphate buffer (pH 7.4) [14].

### Evaluation of lipoperoxidation

Lipoperoxidation products were measured in isolated LDL-c and HDL-c lipoproteins. CDs were obtained after extraction with chloroform: methanol (2:1). A spectrophotometric assay was performed at 234 nm [15]. CDs were quantified using a molar extinction coefficient of 2.7 × 10^4^ M^− 1^ cm^− 1^, and the results are reported as nmol of CD/mg dry weight. LHP levels were evaluated using the assay conditions described by El-Saadani [16]. The calibration curve was obtained using t-butylhydroperoxide 1 mM as the standard. The concentration of LHP was calculated using the molar absorptivity of I_3_ measured at 365 nm (є = 2.46 ± 0.25 × 10^4^ ● M^− 1^ ● cm^− 1^), and the results are expressed as nmol LHP/mg dry weight. MDA levels were evaluated using 1-methyl-2-phenylindole (Sigma-Aldrich, MO, USA) 15 mM for detection at 586 nm. The values ​​obtained are expressed as nmol of MDA/mg dry weight [17]. The dry weight was determined according to the conditions described by Bernal [18].

### Evaluation of protein damage

Protein damage was evaluated in isolated LDL-c and HDL-c. For the nitroblue tetrazolium (NBT) reduction test, we used NBT reagent (Sigma-Aldrich, MO, USA) 0.28 mM and glycine 2 M (pH 10) according to the conditions described by Gieseg [19]. The absorbance was read at 530 nm. The results are expressed as nmol formazan/mg protein. DT levels were determined using a fluorometric assay at 320 nm excitation and 405 nm emission [20]. The results are expressed as nmol DT/mg protein. Protein carbonylation (PC) was determined by treatment with 2,4-dinitrophenylhydrazine (DNPH), which reacts with carbonylated protein derivatives to form stable hydrazones that exhibit an absorption peak at 370 nm [21]. The molar extinction coefficient of 21 × 10^3^ M^− 1^ cm^− 1^ was used to quantify PC content. Values are expressed as nmol PC/mg protein. Protein levels were determined according to the Lowry method [22].

### Quantification of antioxidant defense

PON-I antioxidant enzyme activity was evaluated in HDL-c isolated by the hydrolysis of diethyl p-nitrophenol phosphate (paraoxon-ethyl) (Sigma-Aldrich, MO, USA). The absorbance was quantified at 405 nm. Enzyme activity was calculated according to the molar extinction coefficient of p-nitrophenol, which is 18,053 (mol/L)^− 1^ • cm^− 1^. Activity is expressed as nmol p-nitrophenol/dL Apo-A/min [23]. The total antioxidant capacity assay was measured in plasma by a method based on cupric-reducing antioxidant capacity (CUPRAC) using copper (II) 10 mM and neocuproine reagent 7.5 mM. The reaction mixture was incubated for 20 min at room temperature to quantify non-protein antioxidants and 50 °C to quantify protein antioxidants. The absorbance was determined at 450 nm. A calibration curve was obtained using 2 mM trolox (6-Hydroxy-2, 5, 7,8-tetramethylchromane-2-carboxylic acid) (Sigma-Aldrich, MO, USA) as the standard [24]. The results are expressed as trolox equivalents (TR-equivalent). The TR-equivalent is defined as the nanomolar concentration of a trolox solution with antioxidant capacity equivalent to a 2.0 mM solution of the substance under investigation.

### Statistical analysis

The values obtained are presented as the mean ± standard deviation. Data were analysed using Student’s t-tests using Prism 5.0 (GraphPad, San Diego, CA, USA). The normality of the distribution of quantitative variables was analysed using the Kolmogorov–Smirnoff test. The Pearson correlation coefficient was used to evaluate correlations, and *p* values less than 0.05 were considered significant.

## Results

In this study, we recruited 60 newborns, including 30 newborns from normotensive women (control group) and 30 newborns from preeclamptic women. All of the newborns met the selection criteria for each group. There were no significant differences in gestational age (*p* = 0.1178). Newborns in the PE group and control group were classified as late preterm (according to the classification of the World Health Organization) [25] since they had 38.3 and 37.5 weeks of gestation, respectively. Thus, we can infer that the results of this study are due to alterations induced by PE and not related to prematurity.

For the control group, the percentages of male and female newborns were 40% (*n* = 12) and 60% (*n* = 18), respectively, while for the PE group, the percentages of males and females were 43.3% (*n* = 13) and 56.6% (*n* = 17), respectively.

Compared with newborns in the control group, newborns in the PE group had a decrease of 16.3% in weight and 6.9% in height at birth. Values for the Apgar test at 1 and 5 min were significantly lower in the newborns from preeclamptic women than in newborns from normotensive women (*p* = 0.0203 and *p* = 0.0001, respectively). However, both determinations (first and fifth minutes) in the two groups fell into the healthy range. For the Silverman-Anderson test, which assesses newborn respiratory distress, there was no significant difference between the groups, and the score of both groups (1.65 for the PE group and 1.47 for the control group) indicated mild respiratory distress at birth (range of 1 to 3 points).

GOT and GPT enzyme levels, which indicate liver damage, and creatinine levels, which indicate kidney damage, were not significantly different between the groups.

The lipid profile of newborns in the PE group showed an increase of 18.74% in T-Chol, 30.49% in LDL-c and 30.98% in Apo-B levels. Unlike the levels of TAG, the levels of HDL-c and Apo-A did not show significant differences between groups. Protein and non-protein CUPRAC levels were higher in newborns from women with PE (40 and 28.8%, respectively) than in newborns from women with normotension (Table 1).

**Table 1:** Demographic data and clinical characteristics

Characteristics	Control	Preeclampsia	Statistical significance
Neonates	30	30	–
Gestational age (weeks)	38.3 ± 1.10	37.5 ± 2.18	*p = 0.1178*
Gender: male/female (%)	40 / 60	43.3 / 56.6	–
Delivery: Vaginal birth/Caesarean section (%)	0 / 100	6 / 94	–
Birth weight (g)	*3152 ± 442.2*	*2638 ± 494.7*	*p = 0.0006*
Size (cm)	*49.52 ± 2.11*	*46.07 ± 4.41*	*p = 0.0015*
Apgar score 1 min	*7.87 ± 0.80*	*7.53 ± 0.90*	*p = 0.0203*
Apgar score 5 min	*8.96 ± 0.17*	*8.30 ± 0.73*	*p = 0.0001*
Silverman-Anderson test	1.65 ± 0.57	1.47 ± 0.89	*p* = 0.4378
Respiratory rate (breaths/minute)	49.57 ± 7.94	52.68 ± 6.66	*p* = 0.1620
Heart rate (beats/minute)	143.6 ± 11.03	140.9 ± 12.09	*p* = 0.4375
GOT (U/L)	28.24 ± 8.29	31.37 ± 11.06	*p* = 0.1859
GPT (U/L)	9.05 ± 2.65	8.72 ± 3.18	*p* = 0.6461
Creatinine (mg/dL)	0.72 ± 0.31	0.72 ± 0.12	*p* = 0.9578
T-Chol (mg/dL)	*62.14 ± 19.11*	*73.79 ± 26.78*	*p = 0.0402*
TAG (mg/dL)	33.66 ± 10.80	38.61 ± 15.77	*p* = 0.1325
HDL-C (mg/dL)	31.38 ± 10.01	34.10 ± 14.78	*p* = 0.3676
LDL-C (mg/dL)	*28.76 ± 13.30*	*37.53 ± 16.02*	*p = 0.0188*
Apo**-**A (mg/dL)	88.69 ± 16.40	85.73 ± 30.35	*p* = 0.6047
Apo**-**B (mg/dL)	*23.43 ± 6.79*	*30.69 ± 11.53*	*p = 0.0019*
CUPRAC protein (nmol TR-equivalent/mg protein)	*0.0355 ± 0.0185*	*0.0497 ± 0.0170*	*p = 0.0069*
CUPRAC non-protein (nmol TR-equivalent/mL plasma)	*1.04 ± 0.35*	*1.34 ± 0.36*	*p = 0.0045*

Statistical values were obtained after comparison between newborns from women with preeclampsia (*n* = 30) and newborns from women with normotension (*n* = 30). Values are presented as the mean ± SD. The values in italics are statistically significant (*p*˂0.05)

**Fig. 1:**
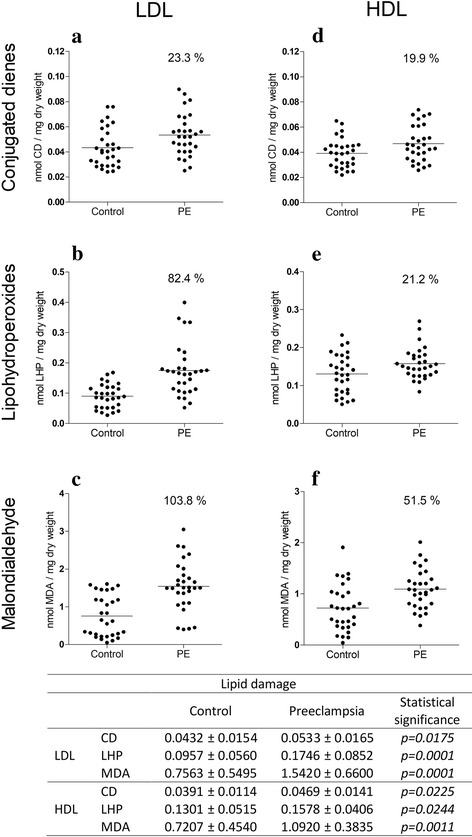
Biomarkers of oxidative damage to lipids in LDL-c and HDL-c. Conjugated dienes (**a** and **d**), lipohydroperoxides (**b** and **e**) and malondialdehyde (**c** and **f**). Statistical values and oxidation levels were obtained after comparison between the group of newborns from women with preeclampsia (*n* = 30) and from women with normotension (n = 30). The data are shown in scatter plots, and the mean population is indicated. In the table, the values are presented as the mean ± SD. The values in italics are statistically significant (*p* ≤ 0.05)

Figure 1 shows the levels of the three biomarkers of lipid damage in LDL-c (Fig. 1a, b and c) and HDL-c (Fig. 1d, e and f). Compared with newborns in the control group, newborns in the PE group showed increased percentages of CD (23.3 and 19.9%), LHP (82.4 and 21.1%) and MDA (103.8 and 51.5%) in LDL-c and HDL-c, respectively. More damage to lipids was observed in LDL-c than in HDL-c. These results were grouped by the severity of lipid oxidation corresponding to the degree of oxidation as follows: DCs: mild oxidative damage, LHP: moderate oxidative damage, and MDA: severe oxidative damage.

**Fig. 2:**
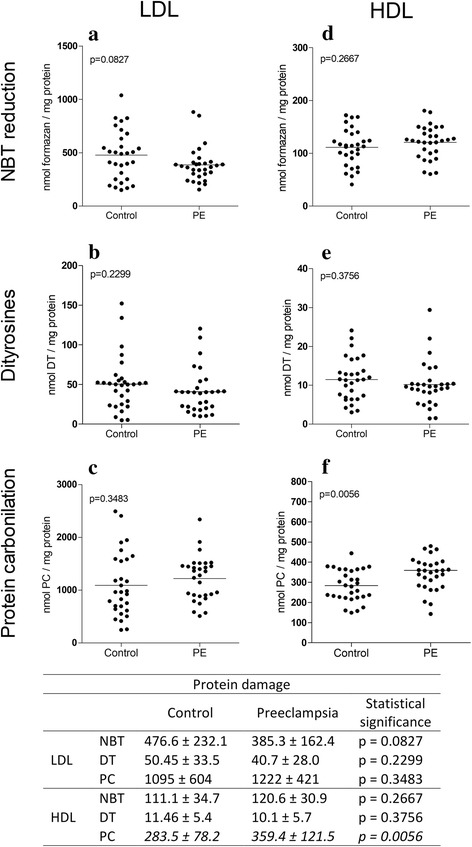
Biomarkers of oxidative damage to proteins in LDL-c and HDL-c. NBT reduction (**a** and **d**), dityrosines (**b** and **e**) and protein carbonylation (**c** and **f**). Statistical values were obtained after comparison between newborns from women with PE (*n* = 30) and newborns from women with normotension (*n* = 30). The data are presented in scatter plots, and the mean population is indicated. In the table, the values are presented as the mean ± SD. The values in italics are statistically significant (*p* ≤ 0.05)

Figure 2 shows the levels of protein damage biomarkers in LDL-c (Fig. 2a, b and c) and HDL-c (Fig. 2d, e and f). Newborns of women with PE exhibited increased levels of PC in HDL-c (26.7%) (Fig. 2f), unlike the same parameter in LDL-c, which was not significantly different. With respect to the NBT reduction and DT levels for both HDL-c and LDL-c, there were no significant differences between the groups.

**Fig. 3:**
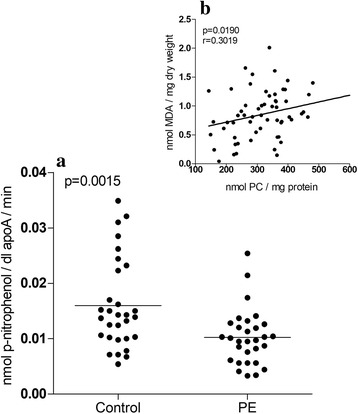
Paraoxonase-I activity. PON-I activity in HDL-c of newborns from preeclamptic women (n = 30) and newborns from normotensive women (n = 30). The data are shown in scatter plots, and the mean population is indicated (**a**). Relationship between MDA in HDL-c and protein carbonylation in HDL-c levels (**b**)

Compared with newborns in the control group, newborns in the PE group showed a 36.2% reduction in the activity of the PON-I antioxidant enzyme (Fig. 3a). MDA and CP in HDL-c were significantly correlated (*p* = 0.0190, *r* = 0.3019) (Fig. 3b).

## Discussion

PE is a main cause of neonatal complications. The study of biochemical and molecular mechanisms, specifically those involving OS and antioxidant status, are key to understanding the origin of clinical complications.

Women with PE exhibit alterations in their lipid profile; however, there are no conclusive reports regarding the lipid profile of their newborns, and in some cases, the results are contradictory. In this study, T-Chol, LDL-c and Apo-B values were increased (Table 1), possibly due to regulation of LDL-c. LDL-c is captured by hepatocytes through the interaction between recognition sites (Apo-B) and their receptors, which is self-regulated by intracellular concentrations of cholesterol. When the cholesterol needs of a cell are met, the cell suppresses synthesis of the LDL-c receptor, which could explain the increased LDL-c values in circulation as well as the increased Apo-B values [26].

In PE, placentation is not carried out properly, and a vascular medium of low capacitance and high resistance is created that generates a state of local hypoxia. This state activates various enzymes, such as xanthine oxidase and nicotinamide adenine dinucleotide phosphate (NADPH) oxidase, and increases superoxide anion (O_2_^•-^) levels, which can form the hydroxyl radical (HO^•^), causing damage to biomolecules, such as lipids and proteins, that make up lipoproteins [27].

**Fig. 4:**
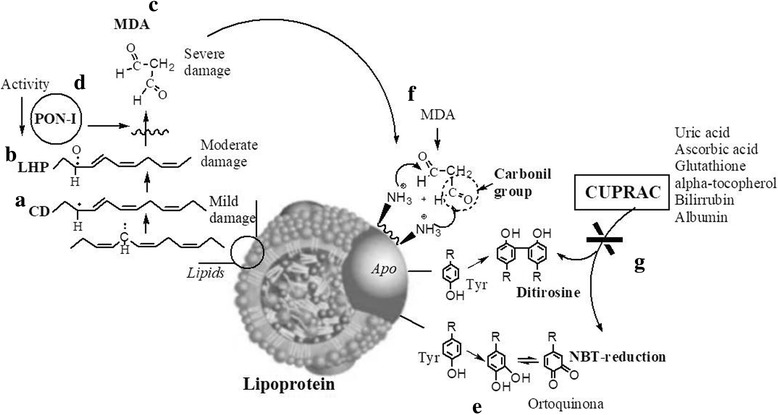
Mechanisms of oxidative damage to LDL-c and HDL-c lipoproteins of newborns from women with preeclampsia. Both lipoproteins have damage to lipids; there are molecular products resulting from lipoperoxidation, such as conjugated dienes (mild damage) (a), lipohydroperoxides (moderate damage) (b), and malondialdehyde (severe damage (c). Lipohydroperoxides can be hydrolysed by the PON-I enzyme. When the activity of this enzyme decreases (d), malondialdehyde levels increase. Additionally, this process can damage proteins through the formation of adducts that induce carbonyl groups (f). Other protein oxidation indicators are through the formation of orthoquinones that can reduce the NBT compound and/or by the formation of dityrosines (e). In newborns from women with preeclampsia, these last two mechanisms of damage do not occur, probably due to the increase in total systemic antioxidant capacity. Non-protein antioxidants, such as uric acid, ascorbic acid, glutathione, and alpha-tocopherol, and protein antioxidants, such as bilirubin and albumin, protect against damage (g)

Generated lipids attacked by HO^•^ undergo molecular rearrangements and form CD (Fig. 4a), LHP (Fig. 4b) and MDA (Fig. 4c). Quantification of lipid oxidation products revealed an increase in isolated LDL-c and HDL-c from the umbilical cord from neonates of women with PE (Fig. 1). We observed that lipid oxidation products are associated with an increase in the percentage of oxidative damage; thus, we propose to classify the damage to lipids into the following stages: DC associated with mild damage, LHP associated with moderate damage and MDA associated with severe damage (Fig. 1). Interestingly, the process of lipid oxidation in fetal lipoproteins was similar to HDL-c and LDL-c isolated from mothers with PE, consistent with a previous report [7].

LHP can be hydrolysed by the PON-I enzyme (Fig. 4d), which is associated with Apo-AI in HDL-c particles. Therefore, PON-I protects against oxidation and is responsible for the antioxidant activity of HDL-c, indicating that the oxidation process continues. However, in this study, the activity of PON-I quantified in the HDL-c of newborns from women with PE was decreased by 36.2% (Fig. 3a). Thus, PON-I was unable to interrupt the oxidative process efficiently because the enzyme was inactivated by OS, leaving it vulnerable to different fractions of HDL-c and LDL-c [28, 29].

Regarding oxidative damage to proteins, the three main protein oxidation indicators are as follows: 1) NBT compound reduction, 2) tyrosine dimer formation and 3) protein carbonylation (Fig. 4e) [7]. The findings obtained with biomarkers of protein damage in HDL-c and LDL-c lipoproteins from newborns from women with PE are impressive because only HDL-c was increased by 26.7% through the protein carbonylation pathway (Fig. 2).

Notably, one protein carbonylation method is the formation of adducts between proteins and reactive aldehydes (alpha-beta unsaturated) by a non-oxidative phenomenon [30], such as MDA [31] (Fig. 4f), which was increased in both HDL-c and LDL-c (Fig. 1). This finding could indicate that the increase in PC is mainly due to lipid oxidation products and not due a direct attack on proteins by free radicals, as indicated by reduced NBT and tyrosine dimer biomarkers. This assertion is corroborated by the positive correlations (*p* = 0.0190, *r* = 0.3019) between MDA and CP in HDL-c levels (Fig. 3b).

Several authors suggest that the intrauterine environment is dramatically impacted by overall maternal health [31]. However, our findings suggest that neonates do not fully share the oxidative modifications that occur in women with PE since these changes encompass important modifications to both lipids and proteins in LDL and HDL particles [7]. Newborns exhibit oxidative modifications at the level of lipids only.

This protective phenomenon could be regulated by systemic antioxidants, including systemic antioxidant capacity for which there are many proteins and non-protein products. Among these factors, we can highlight the role of endogenous fat-soluble vitamins that are found in both plasma and incorporated in HDL-c; these vitamins are involved in the oxidative protection of these particles [24] (Fig. 4 g), which were quantified by the CUPRAC technique. Interestingly, in this study, protein CUPRAC was increased by 40%, while non-protein CUPRAC was increased by 28.8% (Table 1). Another explanation for the lack of oxidative damage to proteins could be an increase in the gene expression of antioxidant enzymes, such as superoxide dismutase (SOD) or glutathione peroxidase (GPx), in healthy neonates [32].

Epidemiological studies demonstrated that the poor quality, restriction and overnutrition of maternal nutrients during pregnancy predispose offspring to a higher prevalence of obesity, insulin resistance, hypertension and heart disease. This phenomenon is referred to as “foetal programming”. Therefore, many diseases of adult life could originate from the foetal and neonatal stages, which are critical periods for the development of organs and systems [33].

Unfortunately, lipids and lipoproteins are not measured routinely during pregnancy, even though women with PE usually have an atherogenic lipid profile and oxidised lipoproteins [7], representing a direct influence on the foetal cholesterol pool. The exposure of the foetus to very high levels of lipoprotein oxidative products could result in programming of foetal arterial tissue with a predisposition to atherosclerosis and cardiovascular risk later in life [34]. Recent discoveries in foetuses, 6-month-old infants and children of mothers with hypercholesterolemia revealed aortic atherosclerosis and early formation of fatty streaks, making these children more prone to metabolic complications in adult life [35]. Therefore, further study of the mechanism of oxidative damage in neonates from women with PE is pertinent since these children may be more susceptible to metabolic complications, inflammatory processes, heart disease, hypertension and atherosclerosis in adult life.

Currently, several drugs are used to regulate hypercholesterolemia during pregnancy; nevertheless, these drugs may alter nutritional status and significantly impact health outcomes in the offspring. These drugs are capable of triggering loss of appetite, nausea, diarrhoea, electrolyte imbalance and changes in glucose metabolism. An approach to improve the quality of life of pregnant women and their neonates is to implement diets rich in antioxidants before and during pregnancy. One proposal is the use of nutraceuticals, which are foods (or part of a food) that provide medical or health benefits, including the prevention and/or treatment of a disease [36]. Nutraceuticals can be used as supplements to influence lipid disorders. Resveratrol may play a positive role in human health via down-regulating proinflammatory conditions or by inhibiting LDL oxidation. Curcumin can prevent macrophage transformation in foam cells by inhibiting scavenger receptor class A [37]. Proanthocyanidins (one of the components of the grape seed) can act on triacylglycerol levels in plasma by reducing their concentration in chylomicrons and very low-density lipoproteins (VLDL-c) [38]. Even supplements with zinc can enhance PON-I activity in patients on hemodialysis and reduce the incidence of cardiovascular disease via a suggested role in prevention of LDL oxidation [39]. Furthermore, the use of nutraceuticals can be considered a helpful tool when standard therapy cannot be adopted due to intolerance. Specifically, nutraceuticals are not a total substitute for all well-standardised pharmacological treatments but can surely improve the outcome of patients with lipid disorders. However, more studies are needed to identify the oxidation pathways of biomolecules and their impact on human health as well as the mechanisms of action of nutraceuticals, appropriate dosages for use in clinical practice and dose-response-related features to adopt these molecules as therapeutics in treatment.

One of the limitations of this study was the small sample size (60 neonates). The cohort of neonates in this study did not present complications at birth; thus, they left the hospital, and we could not provide further clinical follow-up. However, developing future longitudinal studies with larger sample sizes is necessary. Moreover, providing neonates from women with PE with clinical follow-up is important to determine the impact of oxidised lipoproteins on their health during the first years of life.

## Conclusion

Our results showed that the mechanisms of oxidative damage to LDL-c and HDL-c lipoproteins isolated from the umbilical cord of newborns of women with PE worked through lipid oxidation, not protein oxidation. These results allowed us to propose an oxidative profile based on the oxidation levels in each lipid biomarker used. In contrast to a previous study, an increase in protein and non-protein systemic antioxidants was observed. Additionally, the activity of the antioxidant enzyme PON-I quantified in HDL-c particles was decreased. The presence of adequate levels of antioxidants in neonates is key to dealing with the toxic action induced by OS and for the maintenance of cellular and organic homeostasis. The findings in this study are relevant because they contribute to the understanding of the origin of diseases in adult life from the foetal stage. However, it is important to know these mechanisms of oxidative damage in detail. We propose the use of nutraceuticals in addition to pharmacological therapy to improve the quality of life for both mothers and neonates.

## Abbreviations

Apo-A: Apoprotein-A
Apo-B: Apoprotein-B
CD: Conjugated dienes
DNPH: 2,4-Dinitrophenyl hydrazine
DT: Dityrosine
GOT: Glutamic oxaloacetic transaminase
GPT: Glutamic pyruvic transaminase
HDL: High-density lipoprotein
LDL: Low-density lipoprotein
LHP: Lipohydroperoxide
MDA: Malondialdehyde
NBT: Nitroblue tetrazolium
PC: Protein carbonylation
PE: Preeclampsia
PON-I: Paraoxonase-I
TAG: Triglycerides
T-Chol: Total cholesterol
VLDL: Very low-density lipoprotein
